# Vascular Endothelial Growth Factor Receptor-1 Modulates Hypoxia-Mediated Endothelial Senescence and Cellular Membrane Stiffness *via* YAP-1 Pathways

**DOI:** 10.3389/fcell.2022.903047

**Published:** 2022-07-01

**Authors:** Ramcharan Singh Angom, Tanmay Kulkarni, Enfeng Wang, Shamit Kumar Dutta, Santanu Bhattacharya, Pritam Das, Debabrata Mukhopadhyay

**Affiliations:** ^1^ Department of Biochemistry and Molecular Biology, Jacksonville, FL, United States; ^2^ Department of Physiology and Biomedical Engineering, Mayo Clinic College of Medicine and Science, Jacksonville, FL, United States

**Keywords:** hypoxia, senescence, endothelial cells, atomic force microscopy, nano mechanics, hippo pathway

## Abstract

Hypoxia-induced endothelial cell (EC) dysfunction has been implicated as potential initiators of different pathogenesis, including Alzheimer’s disease and vascular dementia. However, in-depth structural, mechanical, and molecular mechanisms leading to EC dysfunction and pathology need to be revealed. Here, we show that ECs exposed to hypoxic conditions readily enter a senescence phenotype. As expected, hypoxia upregulated the expression of vascular endothelial growth factor (VEGFs) and its receptors (VEGFRs) in the ECs. Interestingly, Knockdown of VEGFR-1 expression prior to hypoxia exposure prevented EC senescence, suggesting an important role of VEGFR-1 expression in the induction of EC senescence. Using atomic force microscopy, we showed that senescent ECs had a flattened cell morphology, decreased membrane ruffling, and increased membrane stiffness, demonstrating unique morphological and nanomechanical signatures. Furthermore, we show that hypoxia inhibited the Hippo pathway Yes-associated protein (YAP-1) expression and knockdown of YAP-1 induced senescence in the ECs, supporting a key role of YAP-1 expression in the induction of EC senescence. And importantly, VEGFR-1 Knockdown in the ECs modulated YAP-1 expression, suggesting a novel VEGFR-1-YAP-1 axis in the induction of hypoxia-mediated EC senescence. In conclusion, VEGFR-1 is overexpressed in ECs undergoing hypoxia-mediated senescence, and the knockdown of VEGFR-1 restores cellular structural and nanomechanical integrity by recovering YAP-1 expression.

## Introduction

The survival of the living cells and the maintenance of various biological activities require an adequate O_2_ supply (Taylor, 2008; Yeo, 2019). Reduction in normal O_2_ levels leads to a hypoxic condition, which can give rise to altered pathophysiology. Cellular hypoxia potentially contributes to functional decline during the aging process and is central to the pathophysiology of major diseases, including cancers, coronary heart disease, pre-eclampsia, and kidney disease (Giordano, 2005; Soleymanlou et al., 2005; Fine and Norman, 2008; Eltzschig and Carmeliet, 2011; Chen et al., 2019). For example, reduced O_2_ supply to the cardiac muscles causes a decline in ATP production and cell death within the ischemic region and eventually leads to more extensive MI and worse cardiac function (Depre and Taegtmeyer, 2000; Chiong et al., 2011). Similarly, hypoxic conditions in the brain can also lead to significant morbidities, including cerebrovascular diseases, such as cerebral small vessel disease (CSVD), stroke, Alzheimer’s disease (AD), and vascular dementia (Kivipelto et al., 2001; Gottesman et al., 2010; Gorelick et al., 2011; Montagne et al., 2015). Studies in humans and animal models implicate brain endothelial cell (EC) dysfunction and blood-brain barrier (BBB) failure as early pathological events in cerebrovascular disease. However, the exact mechanisms or initiators of EC dysfunction/BBB failure remain unclear (Zhang et al., 2017; Rajani et al., 2018; Zhang et al., 2019).

In endothelial cells, hypoxia results in the transcriptionally regulated expression of vasoactive substances and matrix proteins involved in vascular remodeling and surrounding tissues (Faller, 1999), including hypoxia-inducible factors (HIFs) (Majmundar et al., 2010; Semenza, 2014). HIFs crosstalk with multiple pathways involved in the regulation of oxygen homeostasis, including angiogenesis (Nakamura et al., 2007; Halligan et al., 2016; D'Ignazio et al., 2017; Chen et al., 2019). Under a hypoxic environment, HIF-1 is stabilized and upregulates various genes, including vascular endothelial growth factors, including VEGF and VEGFR-1, leading to vascular leakiness and neo-angiogenesis (Marti and Risau, 1998; Mukhopadhyay and Datta, 2004; Dvorak, 2006; Nagy et al., 2008). Several laboratories, including ours, have described the diverse roles of VEGF and VEGF receptor pathways involved in vascular hyperpermeability and endothelial homeostasis and dysfunction (Wang et al., 2003; Guangqi et al., 2012; Hoeppner et al., 2012; Simons et al., 2016). Although oxidative stress and inflammatory responses following hypoxia are thought to worsen the cellular response, the exact role of VEGFR-1 signaling in hypoxia-induced EC malformations and remodeling is not well understood and warrants further investigations. Recently we showed that exposure to aging or Alzheimer’s disease-related toxic amyloid-beta (Aβ1–42) oligomers could readily induce a senescence phenotype in human brain endothelial cells (Kulkarni et al., 2019a; Singh Angom et al., 2019) and also increased membrane stiffness in the senescent ECs as measured by atomic force microscopy (AFM) (Kulkarni et al., 2019a). Remarkably, VEGFR-1 Knockdown in ECs was able to prevent endothelial cell senescence mediated by amyloid-beta (Aβ1–42) oligomers, indicating VEGFR-1 dependent signaling mechanisms in this pathway (Kulkarni et al., 2019a; Singh Angom et al., 2019). Indeed, recent transcriptome analysis data has shed more insight into the role of VEGF family members, including VEGFR1 and its ligands in AD pathogenesis. Using single-cell RNA-seq transcriptome analysis, a recent report revealed that ECs in the AD brain showed increased expression of immune markers and angiogenic growth factors and their receptors, including FLT1 (VEGFR1) compared to controls (Mahoney et al., 2019), suggesting a potential link between an abnormal EC angiogenic state and BBB abnormalities seen in AD patients. Similarly, a previous report (using RNA sequencing of AD prefrontal cortex tissue) convincingly demonstrated that increased expression of VEGFR1 and its ligands were associated with a more rapid rate of cognitive decline in AD patients (Lau et al., 2020). And increased ischemic damage was associated with higher expression of VEGFR-1 and its ligand VEGFB (Lau et al., 2020), suggesting a potential link with altered VEGFR-1 expression on EC dysfunction. Thus, to examine whether hypoxia/ischemic conditions can lead to altered EC functions, we exposed endothelial cells to hypoxic conditions and showed that the ECs readily entered a senescence phenotype. We also demonstrate that VEGFR-1 modulates Hippo pathway YAP-1 expression suggesting both VEGFR-1/YAP-1 pathways play an essential role in hypoxia-induced EC senescence, thus providing us with a novel target to pursue the prevention and treatment of EC dysfunction in disease pathogenesis.

## Methods

### Cell Culture and Phenotype Analysis and Hypoxia Treatment

HUVECs were obtained from Lonza. HUVECs were seeded on plates pre-coated with 30 μg/ml collagen type 1 and cultured in EBM medium supplemented with EBM-MV Bullet Kit (5% fetal bovine serum in endothelial cell basic medium with 12 μg/ml bovine brain extract, 1 μg/ml hydrocortisone, 1 μl/mL GA-1000. HBMEC cells were purchased from Cell System (Kirkland, WA, United States) and grown in plates pre-coated with attachment factor (Cell Systems) and cultured in serum-containing medium (Cell Systems). Both HUVECs and HBMECs at early passage (P3) and cells upon ∼70–80% confluency was used for all experiments. The cell morphology was observed under an inverted microscope. For hypoxia treatments, 70–80% confluent HUVECs and the HBMECs were exposed to 1% oxygen for 24, 48, and 72 h using the hypoxia chamber (Modular Incubator Chamber (MIC-101), Billups-Rothenberg, Inc. The Normoxia cells were treated with 20% oxygen.

### Senescence-Associated β-Galactosidase Staining

Cells were fixed with 4% Paraformaldehyde, and the senescence-associated β-galactosidase activity was analyzed according to the manufacturer’s protocol (9860S), Cell Signaling Technology). The cells stained for β-galactosidase were imaged using EVOS microscope at both ×20 and ×40 magnification. For quantification, the image captured at ×20 magnification were utilized (for each analysis, we quantified 200 cells and repeated three times). The cells were imaged under the same condition and counted by using ImageJ software. The percentage of senescent cells was the total number of β-gal positive cells divided by the total number of cells measured (*n* = 200 cells/well) from 3 independent experiments). The images processing was performed using Adobe Photoshop CC 2015.

### Western Blot Analysis

For western blotting, the following antibodies were utilized: VEGFR-1 (Rabbit polyclonal antibody, 2893S), VEGFR-2 (Rabbit polyclonal, 2479S) from Cell Signaling Technology, San Jose, CA, United States; Mouse polyclonal anti-p21(C-19), mouse monoclonal anti-p53 (D-01) antibody was purchased from Santa Cruz. Yap (DH81X) antibodies were purchased from Cell Signaling Technology, San Jose, CA, United States. The anti-β actin antibody was obtained from Sigma-Aldrich. Following the incubation with primary and secondary antibody, the membranes were developed with an enhanced chemiluminescence detection system (Bio-Rad). Total protein lysates of HUVEC cells at different time points of hypoxia treatment were prepared by using the NP40 lysis buffer, and 10 µg of proteins were used for the western blot.

### RT-qPCR

For mRNA expression analysis of the genes, total RNA was extracted using RNeasy Kit (Qiagen). 1 µg of total RNA was reverse transcribed using an iScript cDNA Synthesis Kit (Bio-Rad) as described in the manufacturer’s protocol. The PCR was performed using 0.1 µg of cDNA in a 10 ul of PCR mix containing 500 nM of each primer power sybr master mix (Life Technologies), and the reaction was performed using 7500 PCR system (Applied Biosystems) (40 cycles of amplification at 95°C for 15 s and 57°C for 1 min). The PCR primers used and their sequences are shown in Supplementary Table S1.

### Cell Transfection

For VEGFR-1 and YAP-1 knockdown, HUVECs were seeded in 6-well plates and were transfected with human VEGFR-1 or YAP-1 siRNA (FlexiTube siRNA Premix, obtained from Qiagen. The siRNA transfection was carried out using an Oligofectamine transfection kit (Invitrogen) as per standard protocol. For each gene-targeted, two independent siRNA sequences were tested. The VEGFR-1 siRNA sequences are presented previously (Singh Angom et al., 2019), and the YAP-1 siRNA sequences are shown in Supplementary Table S2.

### Immunofluorescence Staining and Fluorescent Microscopy

The cells were seeded in the chamber slides at a density of 4 × 10^4^/chamber and allowed to settle for 24 h then exposed to hypoxia for how long 72 h. The hypoxia treated cells were stained either Phalloidin (Life technology, A12379) or Yap-1 (1:100) (DH81X) (Cell Signaling Technology, San Jose, CA, United States) according to the manufacturer’s instructions, and DAPI was used to stain the nuclei. The images of the cells were captured by using the Zeiss confocal microscope LSM 880 (United States), and images were captured at a magnification of ×40 by using Zeiss Zen 2 software (United States). The surface area measurements of the cells were performed by using Image J software.

### Atomic Force Microscopy

AFM studies on live HUVECs subjected to various treatments were performed using Dimension Icon Scanasyst AFM (Bruker Corp, Santa Barbara, CA). Morphology and NPs characterization was evaluated using peak force quantitative nanomechanical mapping and nanoindentation techniques, respectively. For each experiment, a 60 mm culture dish containing HUVECs was held firmly onto the AFM stage with the help of a custom-built culture dish holder that enabled accurate tracking of the sample feature by the AFM tip. Morphology studies were performed using scanasyst fluid probe with pyramidal tip geometry bearing a nominal tip radius and spring constant of 10 nm and 0.7 Nm^−1^, respectively. Permanent alteration in cell surface morphology due to sharp and stiff probe characteristics was prohibited by optimizing the scan parameters such as scan rate and peak force setpoint to 100 mHz and 160 pN, respectively.

NPs of HUVECs under various treatments were evaluated using LC-CAL A probe (tip radius of 70 nm and spring constant of 0.1Nm^−1^) bearing spherical tip geometry. As the NPs are not only dependent on the sample but also on the probe characteristics, the spring constant and deflection sensitivity of the probe was optimized by indenting on a hard cell culture dish surface in fluid medium without the cells. Peak force tapping amplitude sensitivity was then calibrated at 1 kHz. Further, the ramping parameters for nanoindentation studies were optimized according to our previously published studies (Kulkarni et al., 2019a; Kulkarni et al., 2019b). Briefly, a loading rate comprising of 4 μm s^−1^ with 2048 samples ramp^−1^ and threshold force consisting of 50 pN was applied that resulted into an accurate force-separation (F-S) curve. The measurement area was restricted to a 500 nm × 50 nm membrane region over the nucleus to minimize the inhomogeneity in the cell membrane and prohibit the influence of substrate on NPs (Engler et al., 2007). Poisson ratio for soft samples such as cells was assumed to be 0.3 for all data analysis purposes (Ebenstein et al., 2009).

Morphology experiments were performed on 8 cells, whereas NPs were evaluated from 16 cells for each treatment unless mentioned otherwise. Both morphology and NPs were evaluated in fluid medium at 37°C, which was maintained using a temperature controller (Lake Shore Cryotronics, Inc. Westerville, OH).

### Atomic Force Microscopy Data Analysis

Bruker’s Nanoscope v1.9 software was used to analyze both the morphology images and F-S curves. Prior to the application of the appropriate contact mechanics model, F-S curves were preprocessed to remove excessive undulations resulting from the soft cantilever’s interaction with fluid medium using boxcar filter. Further, these F-S curves were baseline corrected. We observed non-significant adhesion values from F-S curves for all the treatments including under normoxia. Bearing this observation, we successfully applied Hertzian contact mechanics model to analyze the F-S curves, which is given by,

$$F=\left(\frac{4}{3}\right)\left(\frac{E}{1-\upsilon^{2}}\right)R^{\frac{1}{2}}\delta^{\frac{3}{2}}$$

where, F = Force (from force curve); E = Young’s modulus (fit parameter); υ = Poisson’s ratio; R = Radius of the indenter (tip); δ= Indentation.

All the F-S curves for various treatments including the normoxic condition were analyzed such that the deformation in the cell surface was less than 10% of the feature height, which is an essential criterion in the field of AFM study to disregard the effect of substrate stiffness (Chen, 2014). Origin Pro Lab software was then used to plot boxplots.

### Statistical Analysis

All data presented were representative of at least three independent experiments. Statistical analyses were performed with the Graphpad Prism 7 software (SPSS Inc.). Student T-test was used to compare percentages of β-gal -positive cells, Statistical significance for data (cellular height and plasma membrane roughness) from AFM morphology and nanomechanical properties (Young’s modulus, deformation, and adhesion) determined from AFM indentation experiments.

## Results

### Hypoxia Modulates Vascular Endothelial Growth Factor Receptor Expression and Induces Endothelial Cells Senescence

**FIGURE 1:**
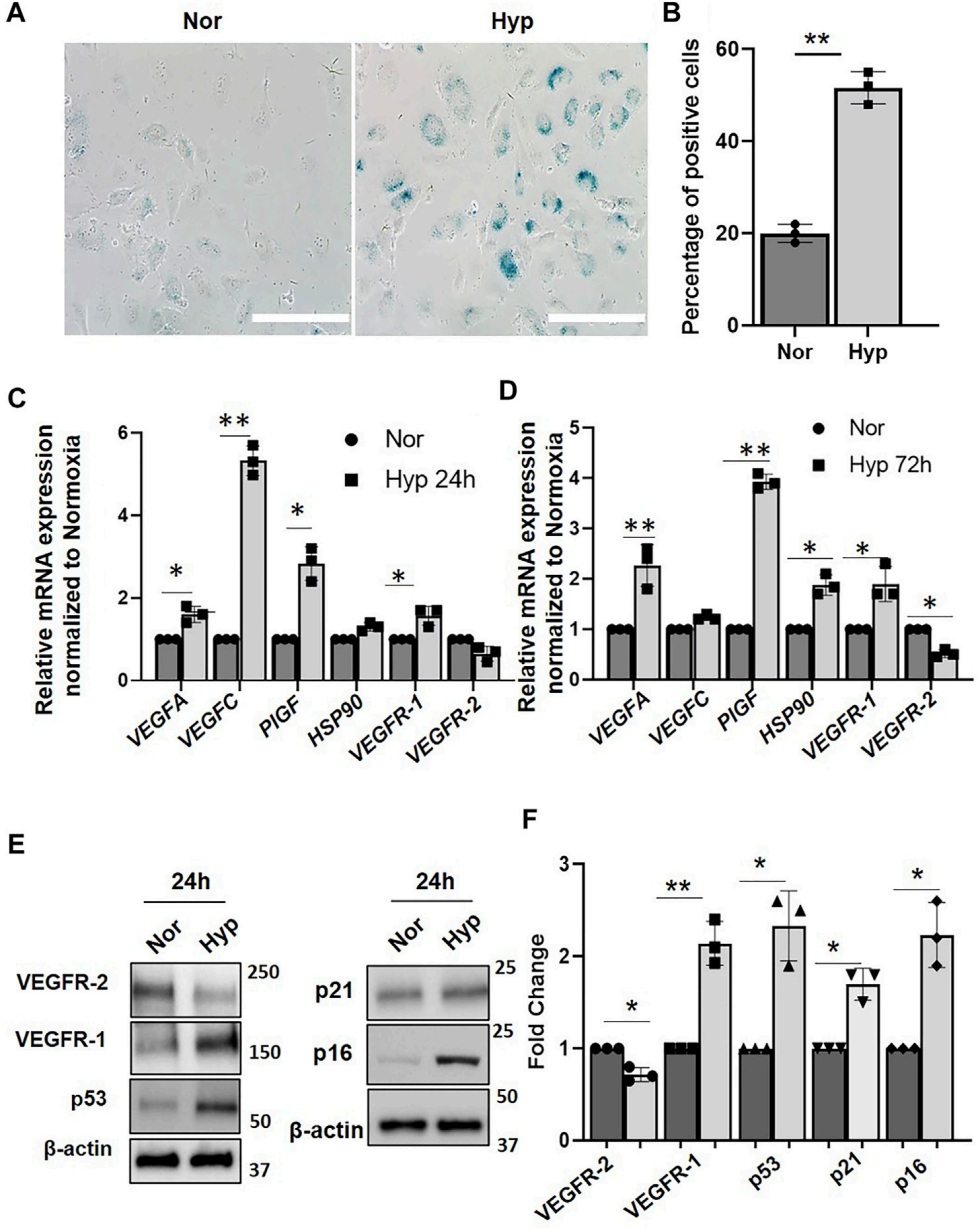
Hypoxia induce senescence phenotype in HUVECs. **(A)** β-galactosidase staining showing HUVECs (Passage 3) treated with normoxia (Nor) and (Hyp) hypoxia for 72 h. **(B)** Quantitative analysis of the β-galactosidase staining from **(A)** (X-axis represents the treatment groups and the Y-axis represents the percentage of cells which are positive for β-galactosidase staining. Relative mRNA expression of VEGF and receptors genes in HUVECs treated with **(C)** normoxia (Nor) and 24 h hypoxia (Hyp 24 h). **(D)** with normoxia and 72 h hypoxia (Hyp 72 h). **(E)** Western blot showing protein expression after exposure to normoxia and hypoxia for 24 h. **(F)** Quantitative analysis of the western blot. (Nor, Normoxia and Hyp, Hypoxia). The error bars represent mean ± SD. These data represent 3 independent experiments. (*, *p* < 0.05 and **, *p* < 0.01, Students *t*-test). (Scale bar = 100 µm).

To investigate whether hypoxia alters protein levels of VEGF receptors in ECs, early passaged (P3) human umbilical vein endothelial cells (HUVECs) were subjected to hypoxia (1% oxygen) for various time points using normoxia (20% oxygen) as control. HUVECs exposed to 72 h of hypoxia (72 h) showed increased (>50%) senescence-associated β-galactosidase staining as compared to that of normoxia (20%) (Figures 1A,B). Quantitative RT-PCR analysis showed increased mRNA levels of angiogenesis factors, including *VEGF-A*, *PlGF*, and *VEGF-C*, in the ECs after 24 and 72 h hypoxia treatment. We also confirmed increased expression of *VEGFR-1* mRNA levels after both 24 and 72 h hypoxia treatment (Figures 1C,D). On the other hand, the *VEGFR-2* mRNA levels were downregulated in response to hypoxia after both 24 and 72 h hypoxia treatment. We also examined the expression of *HSP90,* which is a major regulator of several proteins, including HIF-1α stability (Minet et al., 1999), and observed that the *HSP90* mRNA expression was significantly upregulated (∼50%) when exposed to 72 h of hypoxia. Interestingly there were dynamic changes in specific markers between 24 and 72 h of hypoxia exposure. For instance, *VEGF-C,* which showed a five-fold increase in mRNA levels after 24 h hypoxia, returned to normoxia levels after 72 h (Figures 1C,D), whereas mRNA levels of *HSP90 and VEGFR-1* were more elevated after 72 h hypoxia treatment (compared to 24 h levels), suggesting that hypoxia differentially modulates expression of VEGF family members.

To further confirm the senescence phenotype, we performed western blot analysis to examine the expression of the p53/p21 and the p16/RB pathways which are known to implement the onset of the senescence (Xing et al., 2018). We show significantly increased expression of senescence-associated markers, p16, p21, and p53 protein levels after 24 h of hypoxia compared to the normoxia group (Figures 1E,F), suggesting that hypoxia activates EC senescence pathways. To determine VEGF receptors expression levels following hypoxia, we then performed western blot analysis using EC lysates and showed that VEGFR-1 protein levels were significantly increased after 24 h hypoxia (Figures 1E,F). Quantitation showed a two-fold increase in VEGFR-1 protein levels during hypoxia as compared to that of normoxia (Figure 1F). In contrast, VEGFR-2 protein levels were decreased by ∼40% after 24 h hypoxia (Figures 1E,F).

### Vascular Endothelial Growth Factor-1 Downregulation Inhibits Hypoxia-Induced Endothelial Senescence

**FIGURE 2:**
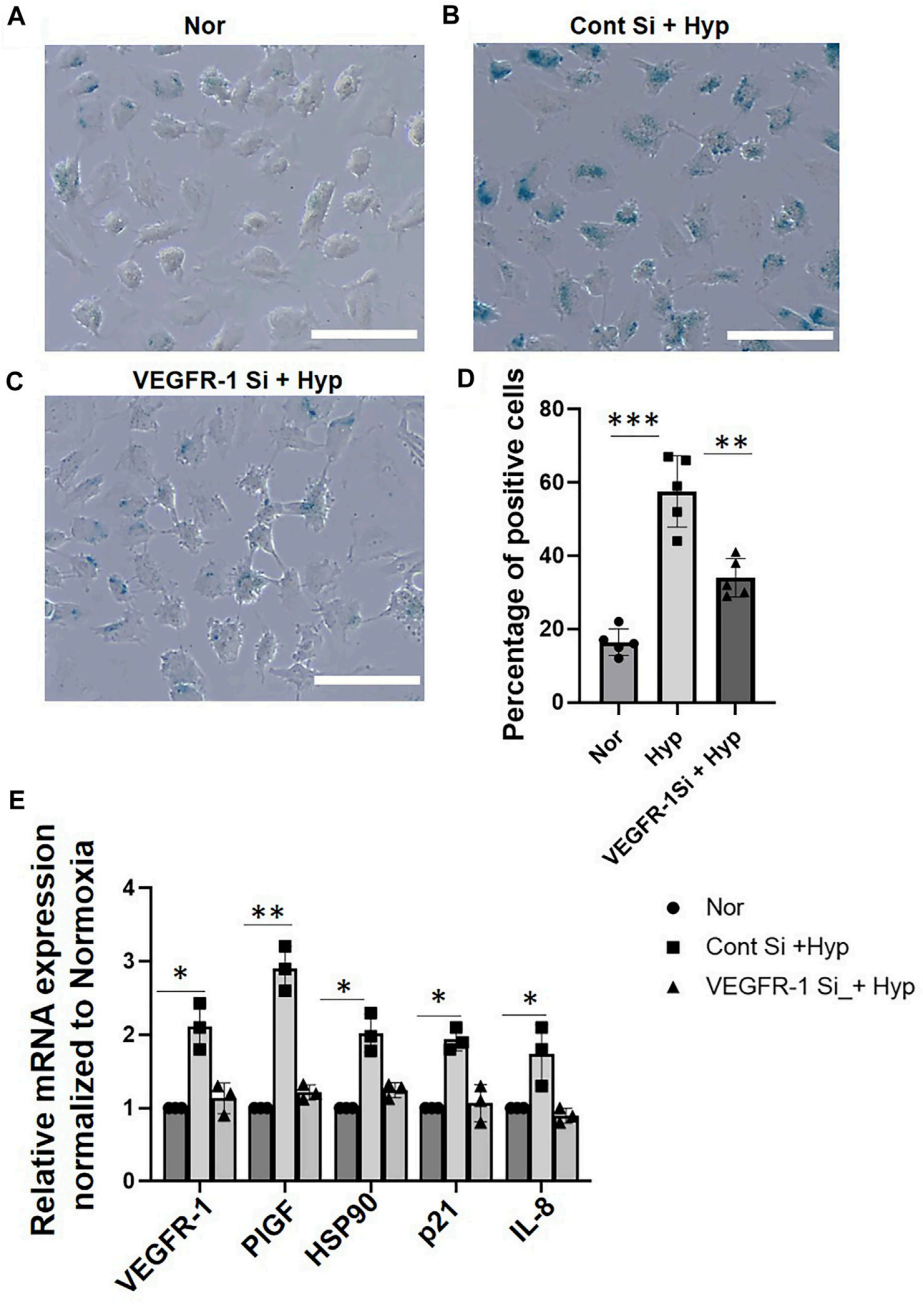
VEGFR-1 knockdown prevents hypoxia induced senescence in HUVECs. **(A–C)** β-galactosidase staining showing HUVECs (Passage 3) treated with normoxia and hypoxia for 72 h with and without VEGFR-1 siRNA, respectively. **(D)** Quantitative analysis of the β-galactosidase staining from **(A–C)** (X-axis represents the treatment groups and the Y-axis represents the percentage of cells which are positive for β-galactosidase staining). **(E)** HUVECs treated with siRNA targeting VEGFR-1 for 24 h, followed by 72 h exposure to hypoxia prevents the upregulation of senescence associated markers. Data shown are from three independent experiments. The error bars represent mean ± SD. (*. *p* < 0.05, ***, *p* < 0.01 and ***, *p* < 0.001, Student’s t-test). (Scale bar = 100 µm).

To further examine the role of VEGFR-1 receptor expression on hypoxia-induced EC senescence, we used siRNA-mediated gene silencing of VEGFR-1 expression as described earlier (Singh Angom et al., 2019). HUVECs were transduced with the VEGFR-1 siRNA for 24 h and were then exposed to hypoxic conditions for an additional 72 h. To access the role of VEGFR-1 in EC senescence, we first performed senescence-specific β -galactosidase staining. We observed that siRNA-mediated VEGFR-1 Knockdown reduced the total number of HUVEC cells displaying the β-galactosidase straining in response to Hypoxia (∼35% positive cells in VEGFR-1 siRNA treated cells vs. ∼55% positive cells in control siRNA treated cells) (Figures 2A–D). Next, we performed qRT-PCR to measure mRNA expression of senescence-associated markers and show that siRNA mediated silencing of VEGFR-1 reduced the mRNA expression of senescence-associated *p21* levels by ∼50% and *IL-8* mRNA levels by ∼55%. (Figure 2E). In addition, siRNA-mediated silencing of VEGFR-1 reduced the mRNA expression of *PlGF* by ∼60%, and *HSP90* by ∼40%, in response to hypoxia treatments (Figure 2E). These results suggest a potential direct role of increased VEGFR-1 expression and signaling events in the onset of EC senescence in response to hypoxia.

### Hypoxia Induces Morphological Alterations in Senescent Endothelial Cells

Previous studies have demonstrated that cellular senescence can induce morphological changes, including enlargement of nuclei (AbuBakar et al., 2014; Kim et al., 2014). In our earlier work (Singh Angom et al., 2019), we had established the classical senescence morphological phenotype, such as the enlarged nucleus in senescent ECs following treatment with amyloid-beta (Aβ1–42) oligomers. In this study, we first performed confocal imaging to examine whether morphological changes were induced in senescent ECs following hypoxia exposure. Confocal analysis of phalloidin stains cells revealed an enlarged morphology in the senescent ECs following 72 h hypoxia compared to normoxia (Supplementary Figures S1A,B). Specifically, we observed an enlarged cell phenotype in approximately 50% of the ECs exposed to 72 h hypoxia compared to ∼10% in normoxia controls (Supplementary Figures S1A–C).

**FIGURE 3:**
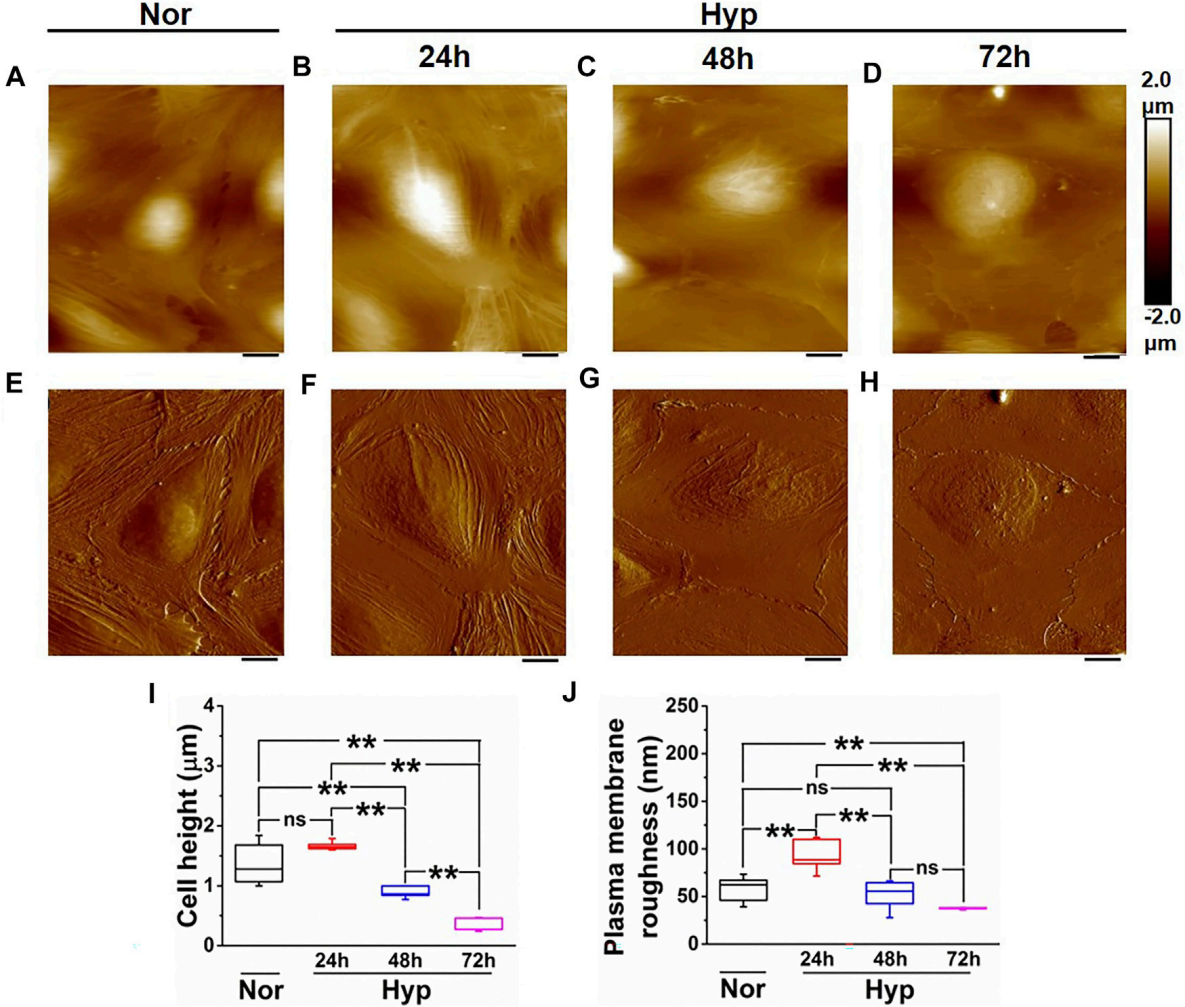
Morphology characterization of HUVECs upon hypoxia treatment. Representative Height image of HUVEC following **(A)** Normoxia. **(B)** 24 h hypoxia. **(C)** 48 h hypoxia. **(D)** 72 h hypoxia. Representative Peak force error image of HUVEC following **(E)** Normoxia. **(F)** 24 h hypoxia **(G)** 48 h hypoxia. **(H)** 72 h hypoxia. **(I)** Average height profile of cells (*n* = 8). **(J)** Average roughness over the nuclear membrane region (*n* = 8). (ns, not significant; **, *p* < 0.01, Students *t*-test) (Scale bars = 10 µm).

We then further characterized the morphological changes in the senescent ECs using AFM to provide a more in-depth analysis of the morphological and structural changes, including membrane ruffling and the enlargement and flattening of cells that may be observed in the hypoxia-induced senescent ECs. In our previous work (Kulkarni et al., 2019a), we demonstrated alterations in membrane ruffling and flattening of ECs treated with amyloid-beta (Aβ1–42) oligomers. Herein, we exposed the HUVECs to hypoxic conditions at different time points (24, 48, and 72 h) and then selected cells for AFM studies that appeared larger and flattened with an enlarged nucleus, both hallmark senescent features. The average cellular height, cell body roughness, membrane ruffling was quantified using high-resolution height profile AFM images. Figure 3A shows a representative height profile image and peak force error image (Figure 3E) of the HUVEC at the normoxic condition. Quantification (Figure 3I) showed cell height with a bulged nucleus (1.35 ± 0.31 µm) and plasma membrane roughness (57.4 ± 12.88 nm). Figures 3B–D shows representative images of height profile, and Figures 3F–H shows representative peak force error images of various hypoxia treatment time points, 24, 48, and 72 h, respectively. In 24 h hypoxia treatment, HUVECs did not show any significant change in their cellular height (1.67 ± 0.07 µm compared to normoxia); however, the plasma membrane roughness was found to be increased (93.36 ± 17.32 nm compared to normoxia). In the case of 48 h (Figures 3C,G) and 72 h (Figures 3D,H) treatments, the cells became progressively flattened, which was confirmed from the average cell height quantification (Figure 3I) with overall height values significantly reduced in 48 h (0.89 ± 0.09 µm) and in 72 h (0.38 ± 0.11 µm) compared to control (1.35 ± 0.31 µm). Moreover, the plasma membrane roughness was observed to become smoother with increased hypoxia treatment times (52.03 ± 14.61) in 48 h, and (37.56 ± 0.98) in 72 h, which was confirmed by the quantification of average cell body roughness as seen from Figure 3J. Altogether, these results demonstrate that the rate of senescence increases with hypoxia treatment times, as evident from the flattened cell morphology and membrane ruffling, and thus can be used as key distinguishing signatures in senescent studies.

### Hypoxia Induces Nanomechanical Alterations in the Membrane of Endothelial Cells

**FIGURE 4:**
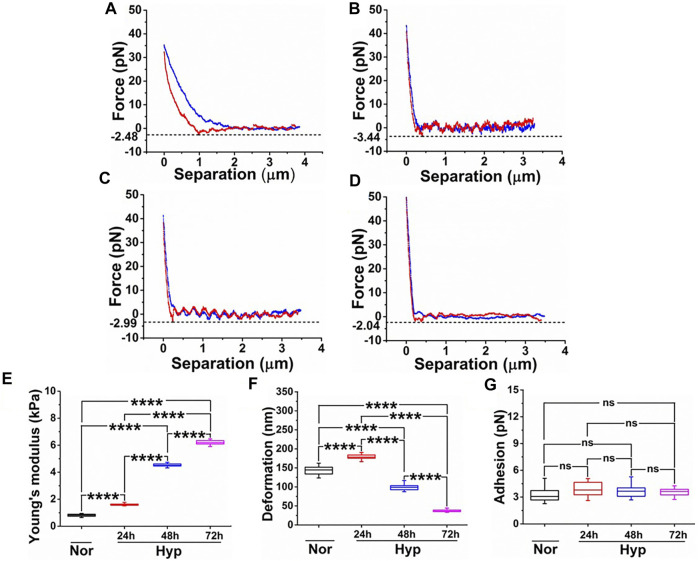
Comparative study of nanomechanical properties of HUVECs derived from the force-separation curves post various treatments. Force-separation curves for **(A)** Normoxia. **(B)** 24 h hypoxia. **(C)** 48 h hypoxia. **(D)** 72 h hypoxia. Nanomechanical properties determined from (*n* = 16) and comprising of **(E)** Young’s modulus. **(F)** Deformation. **(G)** Adhesion. (ns, not significant; ****, *p* < 0.0001, Students *t*-test).

Previously, we have demonstrated that ECs undergoing senescence exhibit unique nanomechanical properties (NPs) of the cell membrane, such as Young’s modulus (a measure of cell membrane stiffness), deformation, and adhesion (Kulkarni et al., 2019a). We employed AFM’s nanoindentation technique to further characterize these NPs in the hypoxia-induced senescent ECs. These NPs are a result of the AFM tip interaction with the cell membrane upon the application of external stimuli, which yields a force-separation (F-S) curve. F-S curve is a plot of force experienced by the tip versus the separation between the tip and the sample (cell membrane). Membrane stiffness is a commonly exploited nanomechanical characteristic using the AFM and is associated with the cytoskeletal actin reorganization. Deformation is a corollary to the membrane stiffness and is a measure of the amount of temporary deformity that occurs in the cell membrane when the tip is in contact with the cell membrane. Adhesion, on the other hand, refers to the pull-off force that the AFM tip experiences while it is retracting from the cell membrane. These parameters cumulatively are used to quantify NPs of various biological entities (Kasas and Dietler, 2008; Redondo-Morata et al., 2014; Kenchappa et al., 2020). In our previous studies, we have shown the importance of optimizing the ramping parameters behind the acquisition of useful and informative F-S curves (Kulkarni et al., 2021; Kulkarni et al., 2022; Kulkarni et al., 2019a). In the present study, we exposed HUVECs to various durations of hypoxia (24, 48, 72 h) and performed nanoindentation using optimized ramping parameters. A representative F-S curve for each treatment is shown in Figures 4A–D. A peak force of 35–50 pN was sufficient for the AFM tip to indent and retract completely, as evident from overlapped trace (blue) and retrace (red) curve with minimal hysteresis. Due to the comparable dimensions of tip diameter and indentation depth, the Hertzian contact mechanics model is employed to analyze these F-S curves. It is evident, the slope of the retrace curve for normal HUVEC (Figure 4A) appears to be gradual compared to hypoxia treatments (Figures 4B–D), indicating that normal HUVECs are softer than the hypoxia treated HUVECs. Within the hypoxia, treated cells corresponding to various time durations, a very subtle difference in the slope of the retrace curve is observed. To confirm these differences, each F-S was individually analyzed using Bruker’s Nanoscope v1.9 software in which the Hertzian contact mechanics model was employed. The average Y_M_ for HUVECs maintained under normoxia was observed to be 0.81 ± 0.09 kPa, which is significantly less compared to the hypoxia treated HUVECs exhibiting an average Y_M_ of 1.61 ± 0.07 kPa, 4.52 ± 0.11 kPa, and 6.25 ± 0.24 kPa at 24, 48, and 72 h respectively, as seen from Figure 4E. This clearly indicates that increasing exposure to hypoxia makes the cells stiffer. As the cell membrane becomes stiffer, with constant applied force, the deformation caused by the AFM tip decreases with an exception for 24 h hypoxia treatment, as seen from Figure 4F. The average deformation for normoxic HUVEC was observed to be 144.42 ± 15.05 nm, which is interestingly lesser than the 24, 48, and 72 h hypoxia treatments exhibiting deformation of 179.54 ± 9.87 nm, 98.76 ± 8.47 nm and 37.56 ± 3.64 nm, respectively as seen from Figure 4F, suggesting that the AFM tip finds an increasing resistance from the cell surface to cause deformation at a constant applied trigger force**.** In this study, however, we did not see any significant changes in the adhesion levels for HUVECs under both normoxic and hypoxic conditions treatments, as seen from Figure 4G.

### Vascular Endothelial Growth Factor-1 siRNA Knockdown Prevents Hypoxia-Mediated Nanomechanical Alterations in Endothelial Cells

**FIGURE 5:**
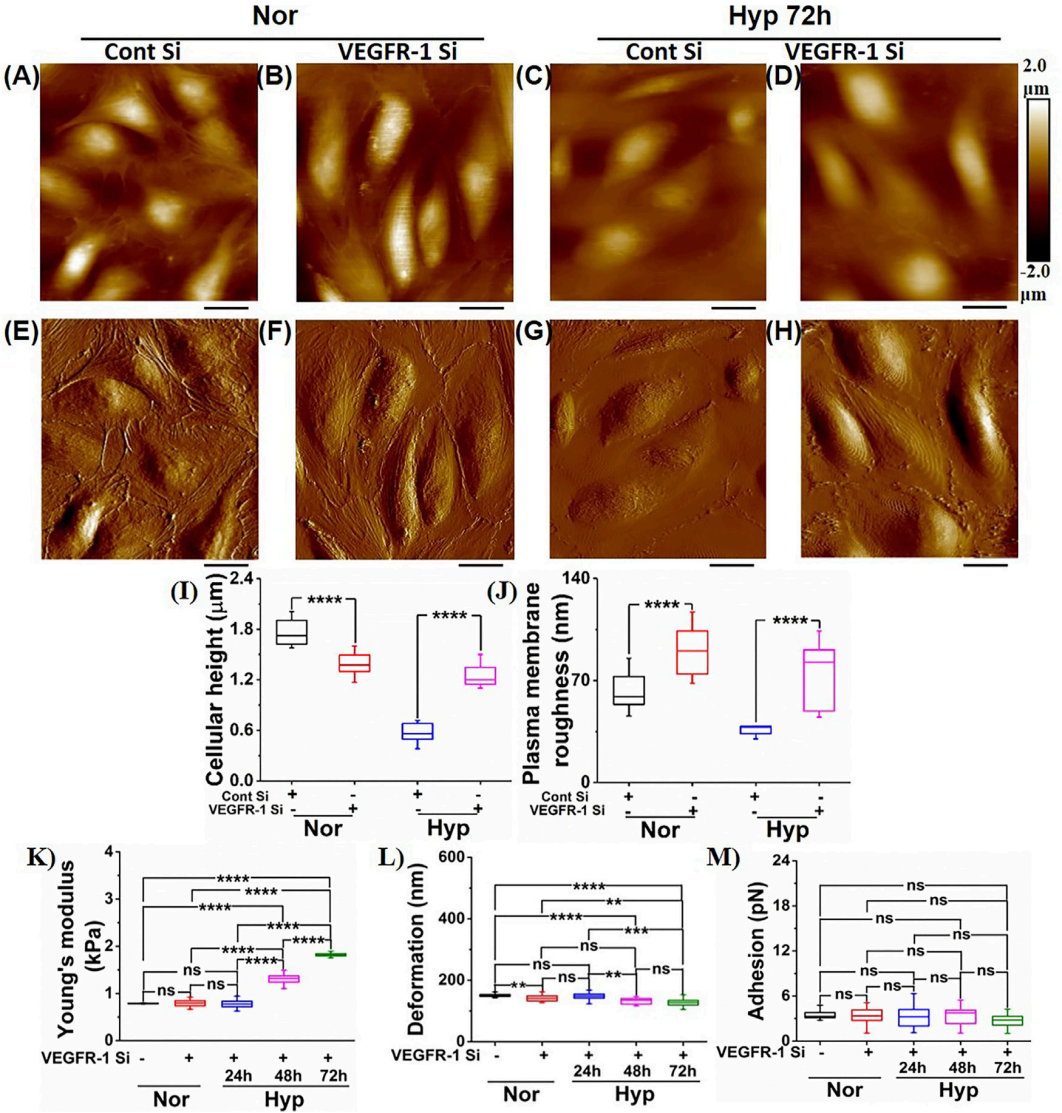
Morphology and nanomechanical properties of HUVECs following VEGFR-1 knockdown. In the presence of normoxia, representative height image of **(A)** Control siRNA. **(B)** VEGFR-1 siRNA. In the presence of 72 h hypoxia, representative height image of **(C)** Control siRNA. **(D)** VEGFR-1 siRNA. In the presence of normoxia, representative peak force error image of **(E)** Control siRNA. **(F)** VEGFR-1 siRNA. In the presence of 72 h hypoxia, representative peak force error image of **(G)** Control siRNA. **(H)** VEGFR-1 siRNA. **(I)** Average height profile of cells (*n* = 8). **(J)** Average roughness over the nuclear membrane region (*n* = 8). Nanomechanical properties of VEGFR-1 siRNA knockdown HUVECs followed by various time duration exposure of hypoxia treatment (*n* = 16 cells). **(K)** Young’s modulus. **(L)** Deformation. **(M)** Adhesion. (ns, not significant; **, *p* < 0.01; ***, *p* < 0.001; ****, *p* < 0.0001, Students *t*-test). (Scale bar = 20 μm)

Previously, we had shown that knockdown of VEGFR-1 in HUVECs and HBMECs prevented senescence induced by Aβ1-42 oligomers (Kulkarni et al., 2019a). Here, we induced hypoxia for 24–72 h in HUVECs treated with both control siRNA and VEGFR-1 siRNA and performed morphology and NPs characterization using AFM. The VEGFR-1 depleted HUVECs with 72 h of exposure to hypoxia presented the most significant morphological changes, as seen from Figure 5. HUVECs treated with control siRNA represented by their height profile image (Figure 5A) and peak force error image (Figure 5E), did not introduce any significant alterations in morphological traits such as cell height (1.76 ± 0.16 µm) and cell body roughness (62.68 ± 13.65 nm) as quantified from Figures 5I,J, respectively when compared to HUVECs under normoxia in Figures 3I,J. Under normoxic conditions, HUVECs treated with VEGFR-1 siRNA preserved the overall cell morphology as seen from representative height profile image and corresponding peak force error image (Figures 5B,F) with a bulged nucleus and more or less similar cellular height (1.38 ± 0.13 µm) and plasma membrane roughness (90.37 ± 17.36 nm) (Figures 5I,J), traits opposite to commonly observed senescence morphology features. Thus, both control siRNA and VEGFR-1 siRNA treatments in HUVECs under normoxic conditions preserve the overall cell integrity. However, HUVECs subjected to control siRNA followed by 72 h hypoxia treatment make the cells appear to be flattened with smoother plasma membrane as observed from their corresponding representative height profile image and peak force error image seen from Figures 5C,G, respectively. Quantification of their cellular height yielded 0.57 ± 0.12 µm, whereas plasma membrane roughness is observed to be 36.17 ± 3.37 nm. On the contrary, VEGFR-1 siRNA treated HUVECs subjected to 72 h hypoxia treatment appeared to delay the plasma membrane roughness (74.25 ± 23.18 nm) over the cell membrane in addition to displaying bulged nuclear region (1.25 ± 0.14 µm), indicating that VEGFR-1 plays a crucial role in hypoxia-mediated senescence in HUVECs evident from their height profile and peak force error images (Figures 5D,H, respectively)**.**

We further quantified and compared the NPs of VEGFR-1 followed by hypoxia treated HUVECs for various conditions, as shown in Figures 5K–M. We also demonstrated that control siRNA and VEGFR-1 siRNA at normoxia conditions do not introduce any significant alterations in NPs, such as cell membrane stiffness and adhesion, as shown in Supplementary Figures S2A–C. NPs of HUVECs subjected to VEGFR-1 siRNA followed by a varying time of hypoxia treatment were determined by analyzing the F-S curves using Hertzian contact model. As seen from Figure 5K, the Y_M_ of HUVEC is similar for normoxia treated (0.81 ± 0.007 kPa) and VEGFR-1 siRNA (0.8 ± 0.08 kPa) treated HUVECs. Membrane stiffness of HUVECs with VEGFR-1 siRNA knockdown subjected to varying durations of hypoxia treatment exhibited stiffness values significantly lower compared to the HUVECs in the absence of VEGFR-1 siRNA treatment and subjected to varying durations of hypoxia as seen from Figures 4E, 5K in which, 24 h exhibited 1.61 ± 0.07 kPa, 48 h exhibited 4.52 ± 0.11 kPa, and 72 h showed 6.25 ± 0.24 kPa (Figure 4E) compared to 24 h exhibited 0.8 ± 0.09 kPa, 48 h exhibited 1.3 ± 0.15 kPa, and 72 h showed 1.82 ± 0.03 kPa (Figure 5K), respectively. In addition, varying significant levels of alterations in deformation values were also observed, as seen from Figure 5L. However, we did not observe any significant change in adhesion values for VEGFR-1 siRNA treated HUVECs followed by varying treatment times of hypoxia as seen from Figure 5M. Comparatively, we also present the quantified NPs of HUVECs subjected to control siRNA and VEGFR-1 siRNA in normoxia (Supplementary Figures S2A–C) as well as hypoxia treatment at varying time durations, as shown in Supplementary Figures S2D–F. From the Supplementary Figures S2A–C, it is evident that control siRNA and VEGFR-1 siRNA treatments in normoxic HUVECs do not alter their NPs. And from Supplementary Figures S2D–F, it is evident that control siRNA-treated HUVECs when subjected to varying time durations of hypoxia treatments, induce senescence and exhibits stiffer membrane with time. However, the decreased stiffness when VEGFR-1 siRNA treated cells are subjected to hypoxic conditions further emphasizes the role of VEGFR-1 in the senescence paradigm.

### Hypoxia Induces Endothelial Cell Senescence *via* YAP-1 Downregulation

**FIGURE 6:**
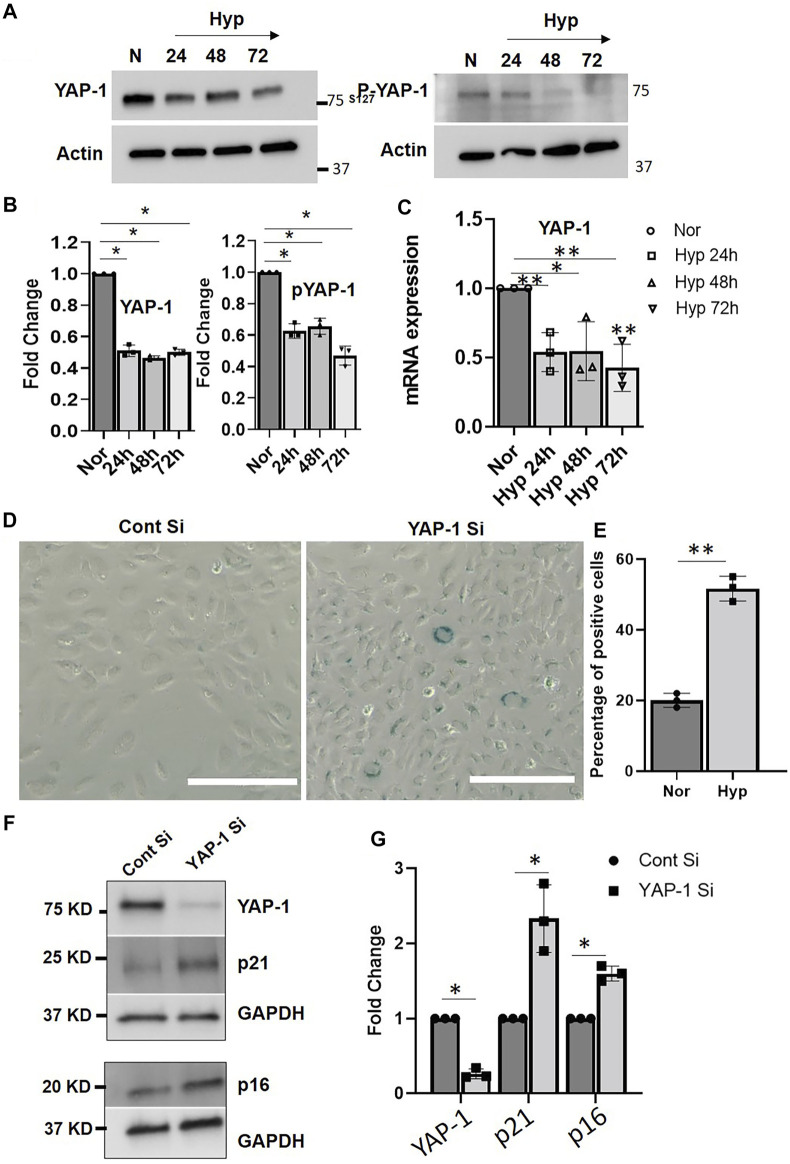
YAP-1 downregulation induces senescence in HUVECs. **(A)** Representative western blot image showing expression of YAP and phospho-YAP when HUVECs were exposed to hypoxia for 24, 48, and 72 h. **(B)**. Quantification showing the fold change of the protein expression. **(C)** mRNA expression of YAP-1 in HUVEC treated with hypoxia for 24, 48, and 72 h. **(D)** β-gal staining of the HUVEC cells after YAP-1 siRNA treatment (Scale bar 200 µm) and **(E)** quantification of the β-gal staining. **(F)** Western blot result showing effect of YAP-1 siRNA knockdown on senescence marker p16 and p21 expression **(G)** Quantification of **(F)** showing significant upregulation of p21 and p16. **(G)** mRNA expressions of YAP1 pathways genes. Error bars indicates the mean ± SD. *, *p* < 0.05, Student’s *t*-test.) (Scale bars = 50 µm). AU = Arbitrary Units *, *p* < 0.05; **, *p* < 0.01, Student *t*-test). (Scale bar = 200 µm).

The YAP-1/Hippo signaling pathway regulates apoptosis and cell survival/proliferation and plays an essential role in mammalian organ size regulation and tumor suppression (Halder and Johnson, 2011; Chen et al., 2019; Fu et al., 2019). Increases in YAP-1 activity are associated with cell proliferation and cancers (Halder and Johnson, 2011), whereas phosphorylation of YAP-1 *via* MST1/2-LATS1/2 results in its cytoplasmic retention, and subsequent degradation, leading to loss of transcriptional activity and induction of apoptosis (Halder and Johnson, 2011). Downregulation of YAP-1 expression in human fibroblast cells and human mesenchymal cells was recently reported to play an important role in cellular senescence (Chen et al., 2019; Fu et al., 2019). To determine the role of YAP-1 signaling in the hypoxia-induced EC senescence, we first examined the expression of YAP-1 in HUVECs exposed to hypoxia. Our data show that hypoxia treatment for 24, 48, and 72 h significantly downregulates both YAP-1 and phospho-YAP-1 protein levels (Figures 6A,B) and YAP-1 mRNA expression levels (Figure 6C) compared to normoxia controls. Next, we examined whether YAP-1 downregulation by itself can induce a senescence phenotype in ECs. As shown in Figures 6C–F, siRNA-mediated YAP-1 knockdown in HUVECs was sufficient to induce senescence. YAP-1 knockdown displayed increased β-galactosidase staining (∼55% positively stained cells) compared to the control siRNA treated cells (∼20% positive staining) (Figures 6C,D). Furthermore, YAP-1 knockdown also upregulated the expression of senescence markers p16 and p21 (Figures 6E,F, and Supplementary Figures S3A,B), suggesting that YAP-1 knockdown can readily induce senescence pathways in ECs.

We then performed AFM morphological studies to examine further the contribution of YAP1 signaling on nanomechanical alterations in the senescent ECs. Supplementary Figure S4A shows a representative height profile image of normoxic HUVECs exhibiting a bulged nucleus with elongated morphological structure, whereas HUVECs subjected to YAP-1 siRNA treatment for 72 h showed flattened nuclear profile with a smooth membrane over the nucleus as seen from Supplementary Figure S4B. Quantification of AFM morphology showed that YAP-1 knockdown cells exhibited cellular height of 0.3 ± 0.06 µm, significantly lower than normal HUVEC (1.62 ± 0.11 µm) as seen from Supplementary Figure S4C. Also, reduced plasma membrane roughness of YAP-1 knockdown HUVEC was observed (36.82 ± 4.54 nm) compared to normal HUVEC (65.27 ± 12.69 nm) Supplementary Figure S4D. Together, these results indicate the YAP-1 signaling is involved in the induction of senescence and consequent nanomechanical alterations seen in the hypoxia-induced senescent ECs.

### Yes-Associated Protein-1/Vascular Endothelial Growth Factor-1 Axis Regulates Hypoxia-Mediated Senescence in Endothelial Cells

**FIGURE 7:**
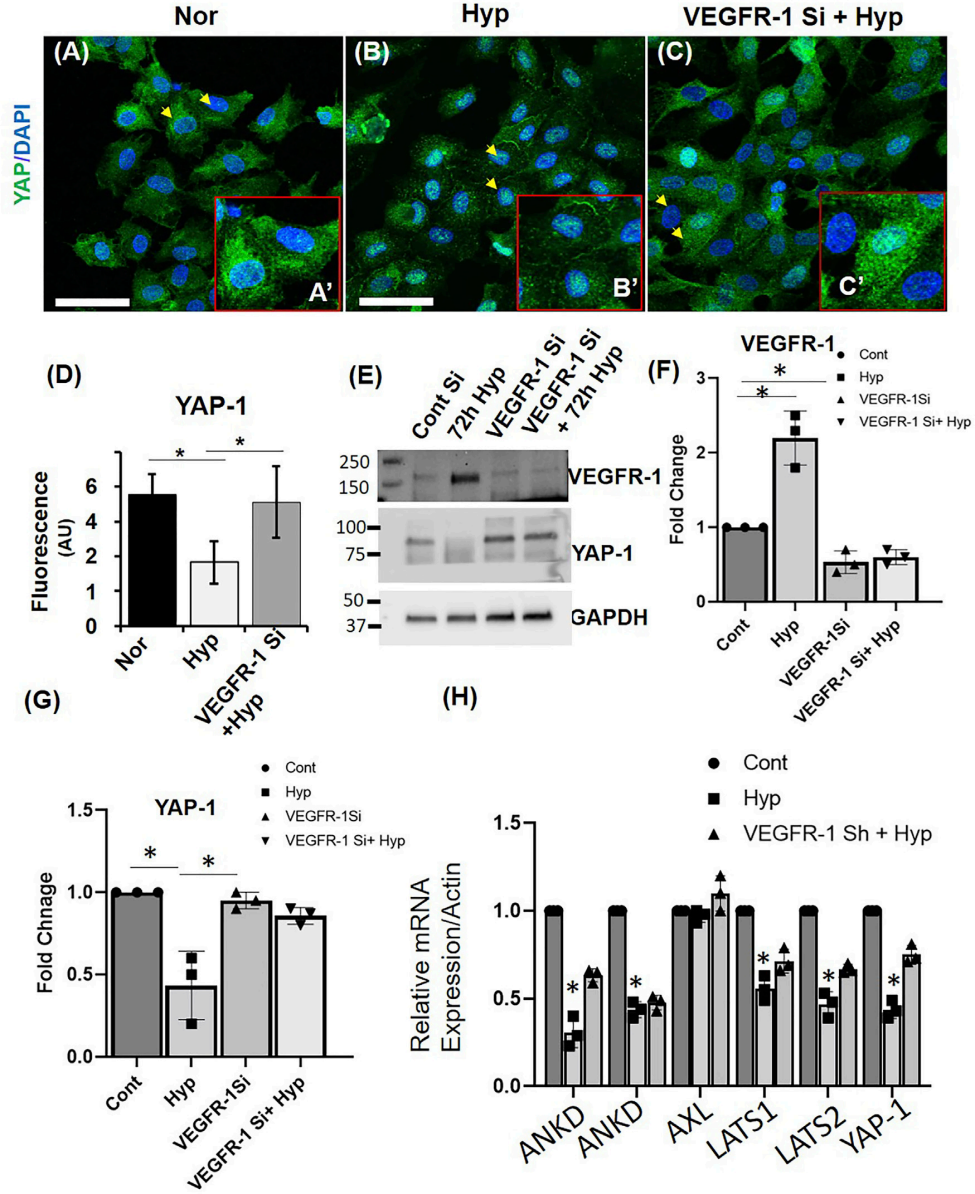
VEGFR-1 Knockdown prevents hypoxia mediated YAP-1 downregulation in HUVECs. **(A–C)** Representative immunofluorescence image showing the expression of YAP-1 in **(A)** Normoxia (Nor), **(B)** hypoxia for 72 h (Hyp) and **(C)** VEGFR-1 knockdown followed by 72 h hypoxia. (Insert shows higher magnification of the cells and the arrowheads indicate the representative cells used.) Green = YAP-1, Blue = DAPI **(D)** Quantification of the fluorescence intensity of YAP-1 staining from **(A–E)** Representative western blot showing VEGFR-1 and YAP-1 expression in ECs following VEGFR-1 siRNA and hypoxia treatment. **(F)** Quantification of the VEGFR-1 expression from the western blot from **(E)**. **(G)** Quantification of the YAP-1 expression from the western blot from **(E)**. **(H)** mRNA expressions of YAP1 pathways genes. Error bars indicates the mean ± SD. *, *p* < 0.05, Student’s *t*-test.) (Scale bars = 50 µm). AU = Arbitrary Units.

To further investigate the effect of Hypoxia on YAP-1 expression, we treated HUVECs with Hypoxia for 72 h. We then examined YAP-1 protein expression by immunofluorescence microscopy and western blots and measured mRNA levels by qPCR. Quantification of immunofluorescence staining showed reduced YAP-1 protein expression in HUVECS (∼50% reduction in hyp vs. nor) in response to Hypoxia (Figures 7A,B,D), and western blot analysis confirmed this reduction [60% in hyp vs. nor (Figures 7E–G)].

A potential link between VEGF–VEGFR-2 signaling and Hippo pathway was recently reported, where it was shown that VEGFR-2 signaling could promote YAP/TAZ activation in endothelial cell angiogenesis (Azad et al., 2018; Elaimy and Mercurio, 2018). We hypothesized a possible interplay between VEGFR-1 and YAP-1 in hypoxia-induced EC senescence. To test this hypothesis, we used VEGFR-1 siRNA to Knockdown the expression of VEGFR-1 in HUVECs and exposed them to hypoxia for 72 h. Quantification of immunofluorescence staining showed that siRNA mediated VEGFR-1 Knockdown rescued the YAP-1 levels in the HUVECs treated with hypoxia for 72 h (60% reduction in hyp vs. 20% reduction in VEGFR-1 siRNA and hyp (Figures 7C,D). This result was further confirmed by western blot analysis (70% reduction in hyp vs. 30% reduction in VEGFR-1 siRNA and hyp (Figures 7E,G). These results suggest a possible role of VEGFR-1 expression and YAP-1 signaling in the hypoxia-induced EC cell senescence. To further confirm these findings, we induced hypoxia (72 h) in additional cell line, namely, human brain microvascular endothelial cells (HBMEC), wherein exposer to 72 h hypoxia showed ∼40% of the cells positively stained for β-galactosidase whereas normoxic cells showed ∼17% staining (Supplementary Figures S5A,B). We then examined the effect of Hypoxia on YAP-1 levels, and then confirmed that hypoxia downregulates the mRNA expression of YAP-1 by 50% in hyp vs. nor in the senescent HBMECs Figure 7H. To further investigate expression levels of YAP-1 regulators, we measure mRNA levels of both upstream mediators (LATS1 and LATS2) and downstream genes, including AXL, CTGF, ANKD, and AREG1, as shown in Figure 7H and Supplementary Figure S6. Hypoxia was found to downregulate the mRNA expression of both LATS 1 and LATS2. Hypoxia also downregulates ANKD and AREG mRNA expression in these cells. Further, VEGFR-1 Knockdown was found to prevent the Hypoxia induced suppression of candidate YAP-1 pathways genes. Interestingly, hypoxia didn’t affect the mRNA expression of AXL, and the VEGFR-1 knockdown didn’t alter the AREG mRNA expression.

## Discussion

In this study, we show that hypoxia conditions induce a senescence phenotype in cultured endothelial cells, including HUVECS and HBMECs. We confirm that hypoxia readily upregulates VEGFA, PlGF, and the expression of VEGFR-1, but we observed decreased levels of VEGFR-2. We also confirmed that hypoxia increased the levels of the senescence-associated markers, including β-gal, and p53, p21, p16 in ECs, further validating the establishment of a senescence phenotype. An important finding of our study is that a Knockdown of VEGFR-1 expression prior to hypoxia exposure delays EC senescence, suggesting an important role of VEGFR-1 expression in hypoxia-related pathogenesis. In agreement with our study, previous reports from other groups also showed that hypoxia increased VEGFR-1 protein levels in ECs derived from various organs in both mice and rats (Tuder et al., 1995; Sandner et al., 1997; Marti and Risau, 1998). Mechanistically, we have previously shown that VEGFR-1 over-expression by itself induces senescence (Kulkarni et al., 2019a; Singh Angom et al., 2019), suggesting that hypoxia-mediated VEGFR-1 expression and signaling may play a key role in the induction of EC senescence. Although some previous studies have shown increased VEGFR-2 expression in response to hypoxia (Waltenberger et al., 1996), the decreased expression of VEGFR-2 levels in our study suggests that VEGFR-2 depletion could also play a role in the induction of senescent phenotype in the ECs.

Secondly, we also demonstrate the involvement of the Hippo-Yes-associated protein/transcription activator YAP-1 signaling, connecting the VEGFR-1 and YAP-1 signaling in the hypoxia-induced ECs senescence. Towards this, we show that 24, 48, and 72 h hypoxia treatment decreased both YAP-1 mRNA levels and protein levels of YAP-1 and phosphorylated YAP-1 in the cultured ECs. In addition, hypoxia treatment of ECs downregulated mRNA levels of both YAP-1 upstream regulators, including LATS1/2 and YAP-1 downstream, gene targets including CTGF and ANKD, suggesting that hypoxia induces transcriptional downregulation of Hippo pathway gene expression in senescent ECs. And siRNA-mediated downregulation of YAP-1 by itself resulted in EC senescence, again confirming that YAP-1 downregulation plays a key role in the induction of EC senescence. Importantly, we show that VEGFR-1 Knockdown normalized YAP-1 expression and delayed hypoxia-induced EC senescence, suggesting a significant role of role VEGFR-1/YAP-1 signaling axis in this paradigm. Previous reports have shown that the Hippo pathway may play a role in vascular cell migration and in angiogenesis during development (Kim et al., 2017; Sakabe et al., 2017; Wang et al., 2017). However, how hippo pathways affect EC functions in other physiological and pathological conditions is not well known. A more recent study (Azad et al., 2018) identified VEGFRs as a potential upstream regulator of the Hippo pathway. In this study, the authors show that VEGFR2 activation by VEGF inhibits LATS and activates the Hippo pathway effectors YAP and TAZ. Furthermore, they showed that the Hippo pathway is a critical mediator of VEGF-induced angiogenesis as either YAP or TAZ KO in ECs inhibited angiogenesis in both *in vivo* and *in vitro* models. Although the authors show that YAP/TAZ knockdown did not induce apoptosis/cell death in the ECs, the exact mechanisms of EC dysregulation seen in these studies was not clear. Our work builds on these previous studies and offers a more precise understanding of the interaction of EC VEGFRs and Hippo pathway signaling in response to pathological conditions and have raised the possibility that YAP-1 might be a downstream signaling component of VEGFR-1, wherein aberrant VEGFR-1 expression/signaling events lead to transcriptional downregulation of YAP-1 and other components of Hippo pathway gene expression, leading to EC senescence, which is a novel finding. Future studies will be needed to further decipher the molecular crosstalk between VEGFR-1 and YAP-1 pathways in this paradigm*.*

We also used AFM as a tool to perform morphological analysis and to examine the nanomechanical property (NP) of the hypoxia-induced senescent ECs. AFM has become a frequent choice for characterizing surface changes associated with both morphology and NPs in ECs (Barbee et al., 1994; Vargas-Pinto et al., 2013; Cabrera et al., 2016), and it is well known that senescence induces morphological changes due to cytoskeletal reorganization and overall enlargement in cell architecture (Le Guilly et al., 1973; Cavallaro et al., 2000; Toussaint et al., 2002; Ben-Porath and Weinberg, 2004). We demonstrate that hypoxia-induced senescent ECs were significantly flattened and exhibited diminished plasma membrane ruffling compared to normal ECs, demonstrating a unique signature of the EC senescence phenotype. Senescent ECs exhibited stiffer membrane as indicated by elevated YM compared to normal ECs, and the stiffness of ECs increased with prolonged exposure to hypoxic conditions. Similar observations were seen in which, a study employing non-interferometric wide-field optical profilometry technique to measure the membrane roughness in living mouse embryonic fibroblasts exhibited dynamics of actin filaments regulates membrane roughness, also confirmed by AFM (Chang et al., 2017). Several studies have demonstrated positive correlation between cell stiffness and actin cytoskeletal rearrangement, and that depolymerization of actin and microtubules lead to softer cells (Rotsch and Radmacher, 2000; Charras and Horton, 2002; Ketene et al., 2012). Prior studies in cancer pathology paradigm, indicated clear trend between cell stiffness and membrane roughness when neuroblastoma cells were treated with paclitaxel (Lee et al., 2016) They observed that a decrease in membrane roughness and an increase in cell stiffness due to translocation of microtubules towards the cell membrane. Similar trend between membrane roughness and cell stiffness was observed in this study. Deformation is a corollary to the observed stiffness. Since we are applying same force level to normoxic and hypoxic cells, stiffer cells exhibited less deformation compared to softer cells. Further, studies have shown that the ability of the cells to deform is associated with higher cell motility and subsequently lower stiffness aiding in cell migration and invasion (Hayashi and Iwata, 2015). Interestingly, we observed an increase in plasma membrane roughness post 24 h hypoxia treatment followed by a consistent decreasing trend post 48 and 72 h hypoxia treatment. The exact mechanism behind such behavior needs to be evaluated in future studies. Significantly, VEGFR-1 Knockdown in ECs followed by hypoxia exposure delayed senescence, where ECs showed morphologically and membrane stiffness characteristics like normal control ECs, suggesting that these morphological alterations and nanomechanical properties can be prevented and reversed. Adhesion is another useful parameter explored by researchers in the field of AFM (Fang et al., 2000; Schaer-Zammaretti and Ubbink, 2003; Puech et al., 2006). However, we did not observe a significant alteration in membrane adhesion properties in these experiments; restricting us to employ Hertzian contact mechanics’ model. Lastly, using AFM, we also demonstrate that YAP-1 knockdown in the ECs showed morphological characteristics reminiscent of senescent cells, such as enlarged and flattened cells with reduced plasma membrane ruffling, again implicating YAP-1 expression pathways in this paradigm. Regulation of YAP expression is caused by factors such as stiff extracellular matrix and shear stress, both relying heavily on cytoskeletal integrity (Panciera et al., 2017). Higher stiffness of the ECM is commonly observed in tumor microenvironment due to elevated integrin signaling, which ultimately results into elevated YAP nuclear localization. This mechano-localized phenomenon could be the result of nuclear flattening inducing pore size change, mechanical protein stability or mechanosensitive nuclear membrane ion channels (Dupont et al., 2011; Humphrey et al., 2014; Low et al., 2014). The role of VEGFR-1 and YAP-1 in hypoxia-mediated senescence from the morphological and nanomechanics perspective is a novel finding, which has not been extensively studied before.

Cellular Hypoxia has been hypothesized to play a major role in the aging process and the pathophysiology of many age-related diseases. Previously, it has been shown that hypoxia affects the cellular metabolism, and other physiological processes including ATP production, Ca^2+^ homeostasis, by impairing the oxygen transport, and results in the generation of reactive oxygen species (ROS) and inflammation (Hassan and Chen, 2021). Hypoxia could lead to alterations in EC functions, ultimately precipitating in vessel malformations. For example, vascular risk factors have been hypothesized to induce BBB dysfunction in AD and vascular dementia (Zlokovic, 2011; Zhao et al., 2015; Nelson et al., 2016; Montagne et al., 2017). VEGFR1 is a member of the RTK family, and although the role of VEGFR2 in endothelial cell survival and proangiogenic signals has been extensively studied, the precise role of VEGFR1 signaling in the postnatal EC function is poorly understood (Fong et al., 1995; Zeng et al., 2001; Carmeliet, 2003). VEGFR1 knock out mice show disorganization of blood vessels and abnormal proliferation of ECs and die at E8.5 to E9.0, suggesting that VEGFR1 may be a negative regulator for proliferation of endothelial cells (Fong et al., 1995). Our data support this notion and suggest that induction of EC senescence *via* VEGFR1- YAP1 signaling events, as a potential mechanism of regulating abnormal EC proliferation during aging and disease process. Our data have raised the possibility that VEGFR-1 is a novel mediator of pathophysiological responses in AD and vascular dementia, leading to EC dysfunction and BBB alterations. In this scenario, vascular risk factors could induce cerebral hypoperfusion due to small vessel constriction or vessel damage, leading to local ischemia/hypoxia conditions and hypoxia-induced BBB leakiness early in the disease process. Continued hypoxia conditions could activate aberrant VEGFR-1 expression and YAP-1 downregulation, leading to EC senescence and neuronal deficits at the neurovascular unit (NVU). Senescent ECs could further damage the NVU; i). by inhibiting EC replacement and regeneration; ii). Inducing changes in tight junction (TJ) protein expression and coverage (Yamazaki et al., 2019), and iii). Secretion of pro-inflammatory cytokines or other factors (Rajani et al., 2018; Barinda et al., 2020) all of which contribute to BBB alterations and vascular malformations.

In summary, our data have identified a novel signaling cascade involving hypoxia-induced aberrant activation of VEGFR-1 and YAP-1 leading to EC senescence and indicate that endothelial cell senescence may be an important early pathological event in the development of hypoxia-induced endothelial cell malformations. Future studies will determine whether rebalancing VEGFR-1 signaling events may delay senescence, inflammation, and nanomechanical alterations of ECs, which may be beneficial in age-related disease models, including AD and vascular dementia.

## Data Availability

The original contributions presented in the study are included in the article/Supplementary Material, further inquiries can be directed to the corresponding author.
