# Supplementary material for: Vascular Endothelial Growth Factor Receptor-1 Modulates Hypoxia-Mediated Endothelial Senescence and Cellular Membrane Stiffness via YAP-1 Pathways
Source: Front Cell Dev Biol. 2022 Jul 1;10:903047. doi: 10.3389/fcell.2022.903047 (PMC9283904; doi:10.3389/fcell.2022.903047)
Supplement: Supplementary file 1 [file DataSheet1.PDF]

Vascular Endothelial Growth Factor Receptor-1 Modulates Hypoxia-Mediated Endothelial Senescence and Cellular Membrane Stiffness via YAP-1 Pathways

Authors: Ramcharan Singh Angom[1], Tanmay Kulkarni[1], Enfeng Wang[1], Shamit Kumar Dutta[1], Pritam Das[1], Santanu Bhattacharya[1,2,] and Debabrata Mukhopadhyay[1,2,*]

Affiliations: [1]Department of Biochemistry and Molecular Biology, [2]Department of Physiology & Biomedical Engineering, Mayo Clinic College of Medicine and Science, FL

*To whom correspondence should be addressed:

Debabrata Mukhopadhyay
Department of Biochemistry and Molecular Biology
Mayo Clinic College of Medicine and Science
4500 San Pablo Road S.
Jacksonville, FL 32224
Email: mukhopadhyay.debabrata@mayo.edu

Running title: Role of VEGFR-1 in hypoxia-mediated endothelial cell senescence

Key words: Senescence, Hypoxia, Nanomechanics, Endothelial cells, Hippo pathway,

Supplementary Figures

Supplementary Figure 1

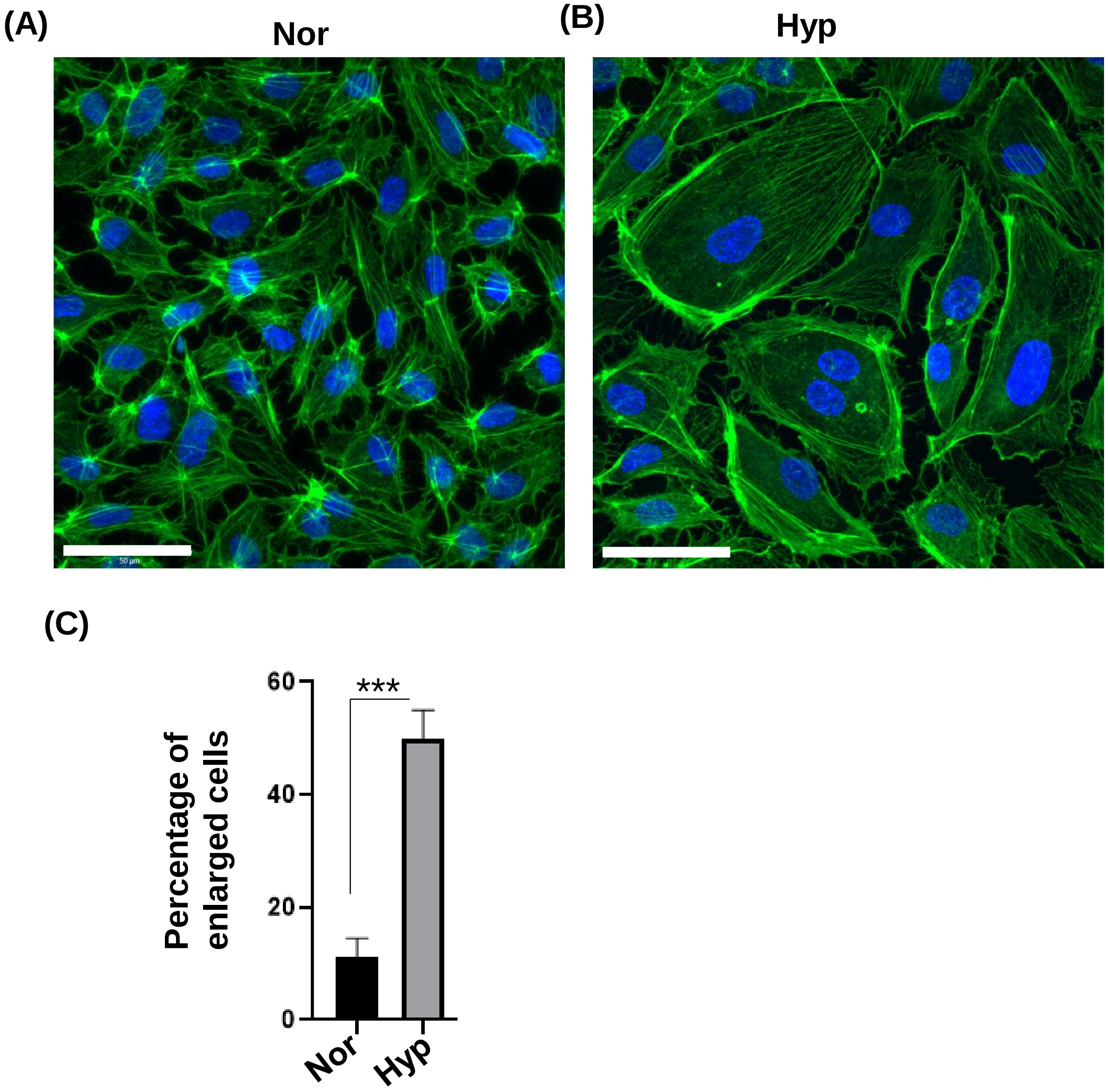

Figure S1. Hypoxia induces both morphological and structural alterations in HUVECs. Representative confocal image of phalloidin stained HUVECs treated with (**A**) Normoxia **(B)** Hypoxia for 72h, **(C)** Quantification of ECs showing the percentage of enlarged cells following hypoxia treatment. The error bars represent mean ± SD. These data represent 3 independent experiments (**, $p < 0.01$ and ***, $p < 0.001$, Students *t*-test). (Scale bar = 50µm).

Supplementary Figure 2

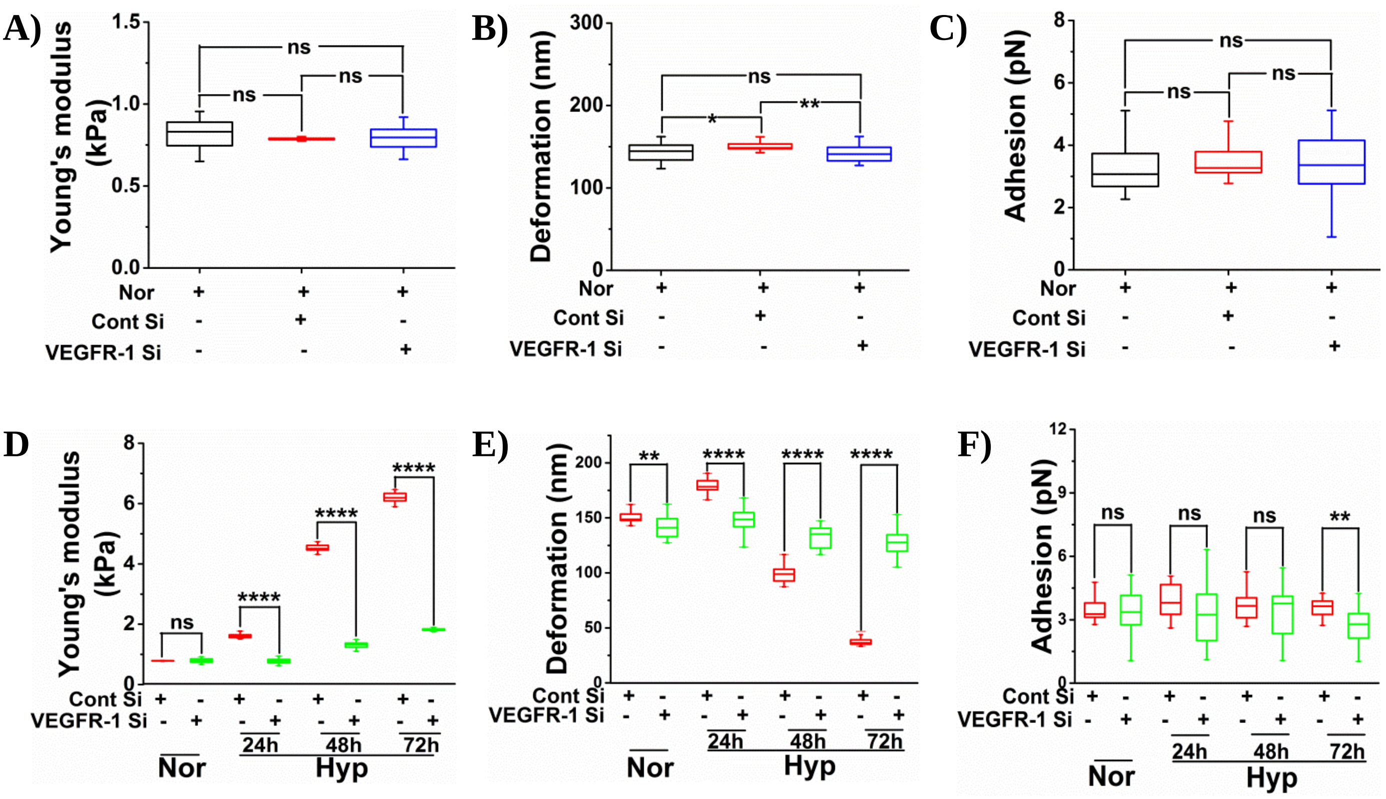

Figure S2. Control experiments to study the nanomechanical properties of HUVECs in the presence of VEGFR-1 siRNA and control siRNA treatment in normoxia. (A) Young's modulus. **(B)** Deformation. **(C)** Adhesion. **Comparison of nanomechanical properties of control siRNA and VEGFR-1 treated HUVECs followed by varying time hypoxia treatment. (D)** Young's modulus. **(E)** Deformation. **(F)** Adhesion. (ns, not significant; *, $p < 0.05$; **, $p < 0.01$; ****, $p < 0.0001$, Students t-test).

Supplementary Figure 3

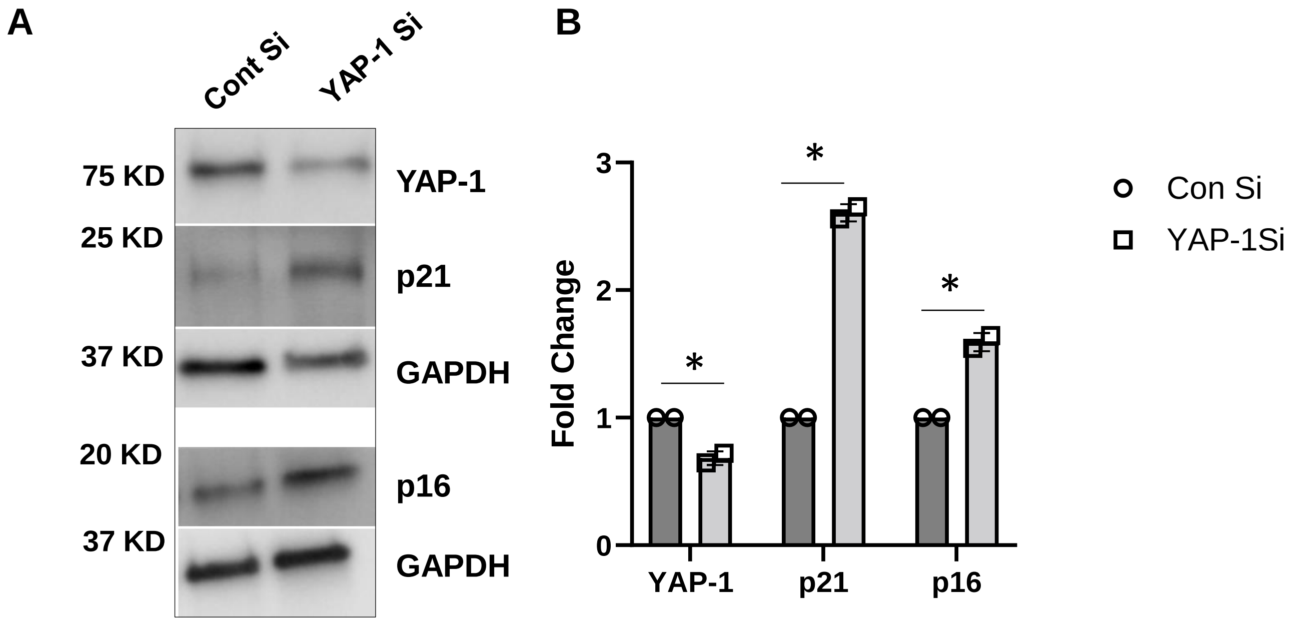

Figure S3. (A) Western blot result showing YAP-1 siRNA-2 mediated YAP-1 knockdown and their effect on senescence marker p16 and p21 expression. (**B)** Quantification showing fold change in proteins.

Supplementary Figure 4

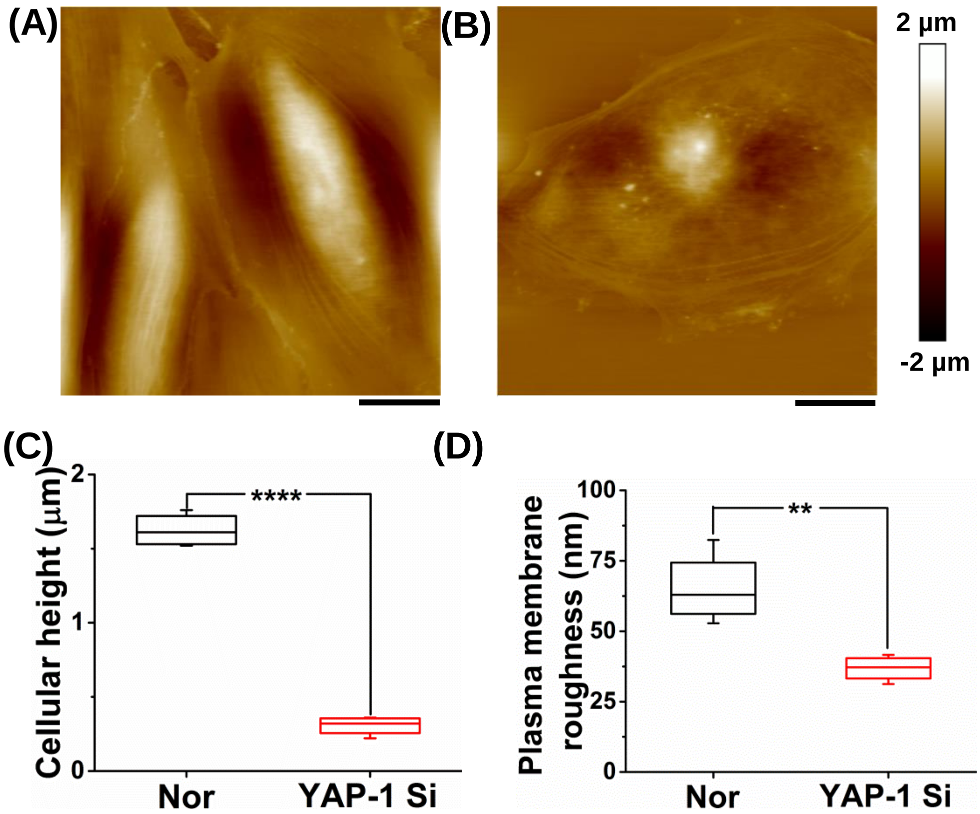

Figure S4. AFM morphology of HUVEC following YAP-1 knockdown. (A) Representative height profile image of untreated HUVEC. **(B)** Representative height profile image of HUVEC after YAP knockdown for 72h acquired by employing Peak Force Quantitative Nanomechanical Mapping (PF-QNM) technique. Quantification of cellular morphology features **(C)** Cellular height and **(D)** Plasma membrane roughness. (**, $p < 0.01$; ****, $p < 0.0001$, Students t-test).

Supplementary Figure 5

(A)

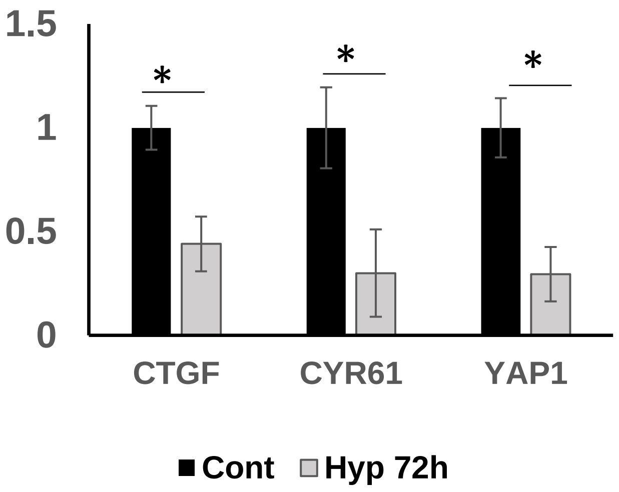

Figure S5. (A). RT PCR showing mRNA expression of YAP-1 and downstream genes in HBMEC cells after hypoxia. The error bars represent mean ± SD (*, $p < 0.05$).

Supplementary Figure 6

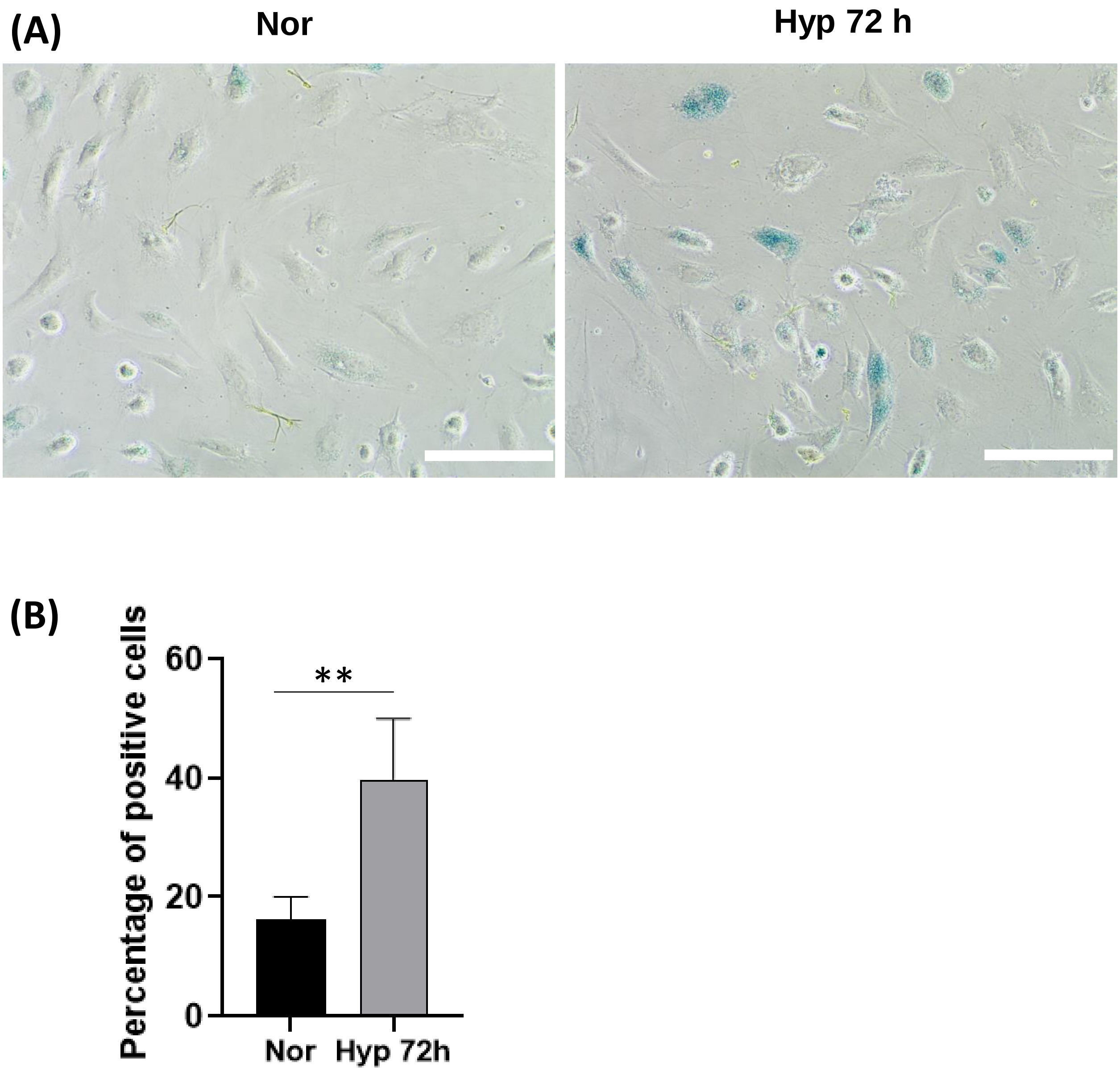

Figure S6. Hypoxia induce senescence phenotype in HBMECs. **(A)** Representative image of β -gal staining of HBMECs treated with normoxia and 72 hours hypoxia. **(B)** Quantification of the cells showing the percentage of b-gal positive stained cells. The error bars represent mean ± SD (**, $p < 0.01$, Students t-test). (Scale bar = 100μm).

Supplementary Table S1

Gene	Forwards	Reverse
IL8	CTTTCCACCCCAAATTTATCAAAG	CAGACAGAGCTCTCTTCCATCAGA
P16	CCAACGCACCGAATAGTTACG	GCGCTGCCCATCATCATG
P21	GAGGCCGGGATGAGTTGGGAGGAG	CAGCCGGCGTTTGGAGTGGTAGAA
P53	CCCCTCCTGGCCCCTGTCATCTTC	GCAGCGCCTCACAACCTCCGTCAT
18S	GTAACCCGTTGAACCCCATT	CCATCCAATCGGTAGTAGCG
VEGF-A	AGGAGGAGGGCAGAATCATCA’	5’-CTCGATTGGATGGCAGTAGCT
VEGF-C	CAGTGTCAGGCAGCGAACAA	’-CTTCCTGAGCCAGGCATCTG
PlGF F	CCGGCTCGTGTATTTATTACCG’	’-GGCAACCACGTTCTCCAGAGC
Hsp90 F	ATGGCTGCTTCCCAGGTGAT GCC,	ACCTCAGGCTCATGACACCAGC
VEGFR-1	TCTCACACATCGACAAACCAATACA	GGTAGCAGTACAATTGAGGACAAGA
VEGFR-2	GCAGGGGACAGAGGGACTTG	GAGGCCATCGCTGCACTCA
Lats 1	CGCTGCTGATGAGATTTGAGTAC	CCACCCTACCCAAAACATCTG
Lats 2	TCTTTGCTCCCCAGGACTTT	GCCAAAGACTTTTCCTGCCA
ANKRD1	AGACTCCTTCAGCCAACATGATG	AGACTCCTTCAGCCAACATGATG
AXL	GTG GGC AAC CCA GGG AAT AT	GTA CTG TCC CGT GTC GGA AAG
AREG	GTGGTGCTGTCGCTCTTGATA	CCCCAGAAAATGGTTCACGCT

Supplementary Table S2

NAME	SEQUENCE
YAP-1 siRNA_1	CACATTAACGACTAGATTAAA
YAP-1 siRNA_2	CACATCGATCAGACAACAACA
